# Toll-Like Receptor 4 Signaling Integrates Intestinal Inflammation with Tumorigenesis: Lessons from the Murine Model of Colitis-Associated Cancer

**DOI:** 10.3390/cancers3033104

**Published:** 2011-08-02

**Authors:** Yasmin Hernandez, John Sotolongo, Masayuki Fukata

**Affiliations:** Department of Medicine, Division of Gastroenterology, University of Miami Miller School of Medicine, Miami, Florida 33101, USA; E-Mails: yasmin.hernandez@hsc.stonybrook.edu (Y.H.); jsotolongo6@gmail.com (J.S.)

**Keywords:** colitis, colitis-associated cancer, bacteria, toll-like receptor, prostaglandin, inflammation, innate immunity

## Abstract

Chronic inflammation has long been implicated as a predisposition for cancer, but the underlying mechanism for how this occurs has remained obscure. Ulcerative colitis (UC) is a chronic inflammatory disorder of the large intestine which is known to be highly linked to colorectal cancer. During chronic inflammation the intestinal mucosa is in a constant cycle of injury and repair resulting in aberrant epithelial proliferation, a process that increases the risk of neoplastic transformation. In particular, the coexistence of commensal flora in the intestine plays an important role in the regulation of mucosal restitution after epithelial injury. It has become apparent that signaling through toll-like receptors (TLRs), the receptor family recognizing pathogen-associated molecular patterns, is crucial to intestinal epithelial proliferation and mucosal restitution. We have recently described two important downstream pathways underlying TLR4-mediated epithelial proliferation in a mouse model of colitis-associated cancer; *i.e.*, cyclooxygenase 2 (COX-2)-mediated production of prostaglandin E_2_ (PGE_2_), and induction of specific ligands for epidermal growth factor receptor (EGFR). These two pathways are closely involved with mucosal levels of PGE_2_ and other prostanoids such as 15-deoxy-delta 12,14-prostaglandin-J2 (15d-PGJ2). Understanding the fine interplay between the TLR signaling and intestinal tumorigenesis in the setting of chronic inflammation can contribute to establishing a novel treatment strategy for inflammation-associated cancers.

## 1. Introduction

Chronic inflammation has been implicated in the development of cancer in many organs including the gastrointestinal tract. Ulcerative Colitis (UC) is a chronic inflammatory disorder within the large intestine, which leads to a constant cycle of injury and repair of the mucosa. UC is one of the diseases that demonstrates a clear link between chronic inflammation and cancer. The intestinal mucosa is in continuous contact with a diverse array of dietary antigens and luminal microbes to which the host maintains a silent state of inflammation. Therefore, disruption of this mucosal integrity has been thought to be the central pathogenesis of uncontrolled inflammation in patients with UC. Although several mechanisms have been proposed to explain how chronic inflammation is linked to cancer development, the exact cause as to how this occurs in patients with UC, especially in the context of host response to intestinal microbes is still obscure.

We have examined molecular mechanisms underlying cancer development during the course of UC by using a mouse model of colitis-associated cancer (CAC) [1,2]. The AOM-DSS model mimics human CAC as it represents repeated cycles of mucosal injury and repair that are associated with increased epithelial proliferation and dysplastic transformation in the large intestine [3,4]. Using the AOM-DSS model, we have previously described that mice deficient in toll-like receptor 4 (TLR4), a pathogen recognition receptor specific for gram-negative bacteria, are resistant to the development of colitis-associated tumors due to decreased expression levels of mucosal cyclooxygenase-2 (COX-2), prostaglandin E_2_ (PGE_2_), and amphiregulin (AR), a ligand of the epidermal growth factor receptor (EGFR). Since exogenous administration of PGE_2_ during the recovery phase of colitis bypasses the protective phenotype of TLR4-deficient mice against colitis-associated tumors, we concluded that TLR4-mediated up-regulation of PGE_2_ during the recovery phase of colitis would be a key for inflammation-associated tumor development in the intestine. The underlying mechanism is that chronic induction of mucosal PGE_2_ forms a positive feedback loop leading to sustained up-regulation of COX-2 in macrophages and AR release from epithelial cells. Both PGE_2_ and AR induce epithelial cell proliferation through EGFR activation and uncontrolled activation of this pathway is known to result in the development of cancer. Elucidating how TLR4-mediated regulation of epithelial proliferation leads to cancer will provide a novel insight into the pathogenesis of inflammation-induced tumorigenesis in the intestine.

## 2. Regulation of Intestinal Epithelial Proliferation

Increased epithelial cell proliferation has been implicated in the development of colorectal cancer [5,6]. Epithelial cells in UC mucosa tend to be hyper-proliferative, which is known to predispose to genetic mutations thereby increasing cancer risk [6,7]. The epithelial lining of the gastrointestinal tract is regularly replaced every two to seven days. In addition to the physiological cycle of regeneration, epithelial turnover can be facilitated as a result of injuries or inflammation and is regulated by the crypt stem cell niche and the surrounding mesenchymal cell populations [8,9]. Subepithelial myofibroblasts are known to play a crucial role in the regulation of epithelial differentiation and proliferation by secreting tropic factors [10,11]. Recently, subepithelial macrophages have been shown to regulate the differentiation of colonic stem cells and epithelial proliferation in response to intestinal microbes [12]. This regulation of epithelial proliferation is implicated in TLR signaling through an adapter molecule MyD88 and consequent expression of Ptgs2, a gene encoding COX-2 and a precursor for PGE_2_ [13]. Therefore, infiltrated macrophages and activated myofibroblasts may be involved in the pathogenesis of CAC by inducing aberrant proliferation of intestinal epithelial cells during chronic inflammation, which is dependent upon TLR signaling in response to luminal microbes.

## 3. Mechanism Underlying TLR4-mediated Intestinal Tumorigenesis

Although deregulated proliferation is involved in inflammation-associated cancer, currently little is known about the nature of this regulation. Following mucosal injury, an inflammatory response is generated leading to up-regulation of many pro-inflammatory mediators. Many cytokines, chemokines, and prostaglandins have been identified as having tumorigenic potential forming a microenvironment that favors tumor growth [14,15].

Nuclear factor kappa B (NF-κB), a pro-inflammatory transcription factor, has long been identified as a crucial factor in developing inflammation-associated cancer because of its tight regulation of cell survival and anti-apoptotic genes [16-18]. Inhibition of NFκB activation by IκB kinase inhibitors has significantly suppressed tumorigenesis in a murine model of CAC [19]. However, strong inhibition of NF-κB may be detrimental as the mice deficient in NFκB die *in utero*, presumably due to its multiple roles involving diverse upstream signaling [17,20]. Although the definitive upstream signaling that is responsible for NFκB activation during development of CAC has not been identified, TLR signaling may be involved in sustained activation of NFκB in the context of colitis-associated tumorigenesis [18].

**Figure 1.**
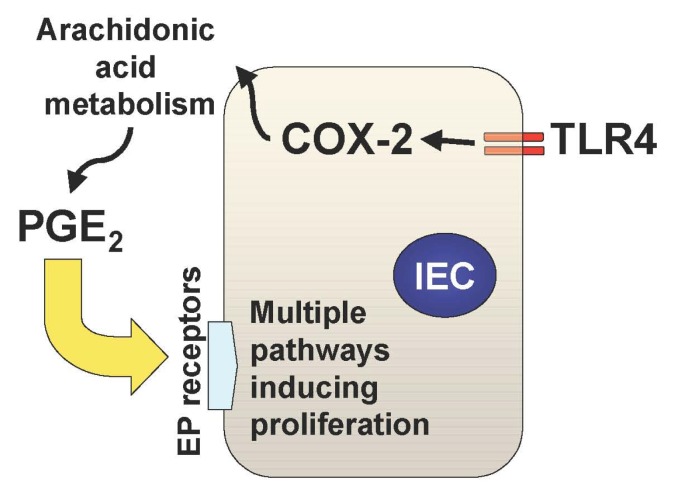
The TLR4-COX-2-PGE_2_ axis. In the setting of intestinal inflammation, epithelial TLR4 is up-regulated. TLR4 signaling induces and stabilizes COX-2. COX-2 acts on arachidonic acid to generate PGE_2_ through a series of metabolic cascades. PGE_2_, through its receptors (EP receptors), can activate downstream signaling molecules that are associated with proliferation in intestinal epithelial cells.

In addition to the TLR-mediated NF-κB activation in the intestine, we have recently described two important downstream pathways involved in TLR4-mediated epithelial proliferation in the intestine. First, TLR4 signaling induces COX-2 expression in lamina propria macrophages and to a lesser extent in epithelial cells, which results in up-regulation of mucosal PGE_2_ (Figure 1) [21]. This up-regulation of PGE_2_ appears to be required for mucosal restitution in response to epithelial injury [21]. However, sustained activation of this TLR4-COX-2-PGE_2_ axis may result in aberrant epithelial cell proliferation and thus lead to colitis-associated tumor development [1,2].

**Figure 2.**
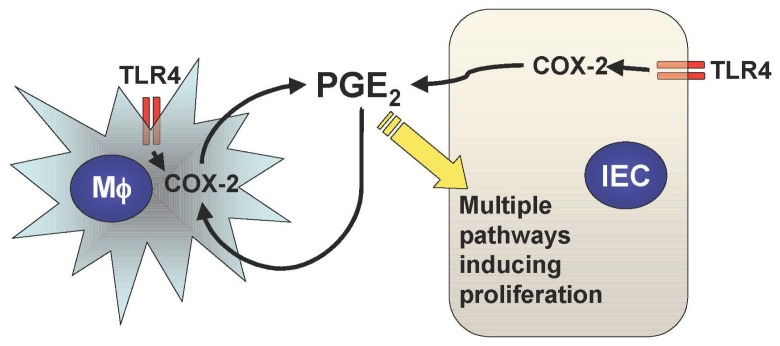
COX-2 and PGE_2_ form a positive feedback loop within the intestinal mucosa. Mucosal TLR4 expression is increased in chronic intestinal inflammation and PGE_2_ is synthesized by COX-2 induction. The PGE_2_ in turn stimulates COX-2 production in mucosal macrophages (Mɸs) which causes further release of PGE_2_, which forms a positive feedback loop thus inducing tumorigenesis.

Mucosal TLR4 expression has been shown to be up-regulated in the setting of chronic inflammation in human and mouse models of UC [1,22-25]. Both COX-2 expression and PGE_2_ production are also increased in UC mucosa, although upstream signaling leading to their induction has not been described [26-28]. Since TLR4-deficient mice are unable to induce mucosal COX-2 expression and subsequent PGE_2_ production in response to intestinal mucosal injury, deregulated TLR4 signaling may be responsible for the increased COX-2 expression and PGE_2_ production in UC mucosa [21]. Furthermore, we have found that increased mucosal levels of PGE_2_ form a positive feedback loop with COX-2 induction within intestinal mucosa, causing sustained epithelial proliferation (Figure 2) [2]. Therefore the continuous overdrive of the epithelial TLR4-COX-2-PGE_2_ axis during the chronic phase of colitis may be the key factor in the pathogenesis of CAC.

**Figure 3.**
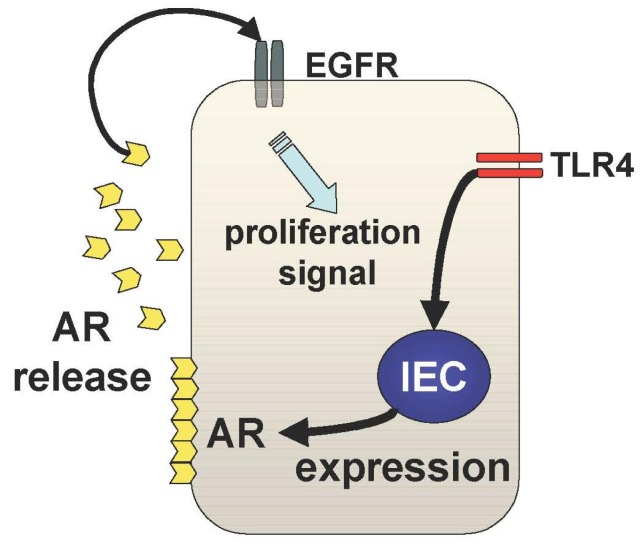
The TLR4-AR-EGFR axis. TLR4 signaling in intestinal epithelial cells induces the expression and release of AR, an EGFR ligand. Released AR binds to the EGFR in an autocrine and paracrine fashion. EGFR signaling is associated with increased proliferation of the epithelial cells.

The other mechanism underlying TLR4-mediated epithelial proliferation is through the TLR4-AR-EGFR pathway (Figure 3). We and others have shown that mucosal expression of EGFR ligands, AR and epiregulin, are regulated by TLR4 signaling during colitis [29,30]. Interestingly, TLR4 signaling induces these EGFR ligands with different kinetics throughout the course of colitis [29]. Epiregulin is expressed during the acute phase of colitis and AR is up-regulated in the chronic period of colitis suggesting distinct roles of these ligands in mucosal repair. It is likely that mucosal AR production is more responsible for intestinal tumorigenesis than epiregulin although both ligands induce activation of the same receptor. In fact, mice deficient in epiregulin are more susceptible to chemically-induced colitis but similarly induced intestinal tumors in Apc/Min model compared to WT mice [31].

In TLR4-deficient mice, neither AR nor epiregulin is expressed following mucosal injury, which is consistent with their phenotype, which includes a defective recovery from colitis [32]. Therefore, induction of these EGFR ligands in response to mucosal injury or inflammation is necessary to repair the epithelial defects; however, sustained production of AR results in colitis-associated tumorigenesis. This idea is supported by the fact that AR expression is increased in UC mucosa and colorectal cancer tissue [33].

The question that now arises is how and why there is selective and sustained production of AR in the pathogenesis of CAC. The answer to this is still obscure, but our data, which demonstrates an induction of mucosal AR by exogenous PGE_2_ administration in TLR4-deficient mice, indicates that sustained mucosal production of PGE_2_ may at least be part of the underlying mechanism by which sustained production of mucosal AR during TLR4-mediated tumorigenesis is involved in the pathogenesis of CAC.

## 4. TLR4 Signaling Mediates Cell Proliferative and Anti-Inflammatory Prostaglandins: Their Role in Developing CAC

Intestinal homeostasis is characterized by a balance of pro-inflammatory and anti-inflammatory mediators in response to luminal microbes and mucosal damage. The arachidonic acid metabolites are known critical mediators in maintaining this balance during the healing process of damaged mucosa in the intestine [34,35]. Intestinal prostaglandin synthesis is induced by the expression of COX-1 and COX-2, and the physiological balance between inflammatory (cell-proliferative) PGE_2_ and anti-inflammatory 15d-PGJ2 maintains mucosal homeostasis. Although mucosal production of PGE_2_ has been shown to be up-regulated in patients with UC and CAC, little is known about mucosal 15d-PGJ2 production in those patients [28,36].

In the murine CAC model, we have described that TLR4 signaling is a dominant regulator of mucosal COX-2 expression and thus plays an important role in balancing mucosal production of PGE_2_ and 15d-PGJ2 during both the acute and chronic phases of colitis [2]. Compared to PGE_2,_ which acts as an acute inflammatory mediator and inducer of epithelial proliferation, 15d-PGJ2 is known to suppress inflammatory gene induction and instead induces cell apoptosis [37,38]. We found that TLR4-mediated induction of COX-2 results in production of both PGE_2_ and 15d-PGJ2 during the acute phase of colitis [2]. However, the mucosal level of 15d-PGJ2 returns to baseline during the chronic phase of colitis despite continuous induction of COX-2 and PGE_2_. Therefore, an additional factor that negatively regulates mucosal production of 15d-PGJ2 and/or a mechanism by which PGE_2_ is selectively induced during the chronic phase of colitis is suspected. Further elucidation of the detailed mechanism which balances the mucosal production of PGE_2_ and 15d-PGJ2 during acute and chronic phases of colitis may directly contribute to establishing a novel strategy to prevent development of CAC in patients with UC.

## 5. Concluding Remarks

TLR4 signaling integrates inflammatory signaling and tumorigenesis through the two unique downstream pathways; the COX-2-PGE_2_ and the AR-EGFR axes. These pathways are necessary to repair the damaged mucosa in the intestine, but continuous activation of these pathways may cause tumor development through amplification of COX-2. Two types of prostaglandins can be induced by COX-2 expression, *i.e.*, PGE_2_ and 15d-PGJ2, which trigger epithelial proliferation and apoptosis, respectively balancing mucosal integrity during inflammation. An imbalance of these prostaglandins resulting in PGE_2_ predominance seems to be involved in CAC pathogenesis and can be induced by abnormal TLR4 signaling.

There are many facets of TLR4-mediated tumorigenesis that need to be elucidated prior to establishing efficacious clinical strategies to prevent CAC. In addition to the direct contribution of epithelial TLR4 signaling to neoplastic development, the contribution of TLR4 signaling to anti-tumor immunity needs to be evaluated in order to establish treatment for CAC. Mice deficient in MyD88, a major downstream molecule of most TLRs, and Interleukins 1 and 18, demonstrate conflicting phenotypes in the context of intestinal tumorigenesis depending on the presence or absence of background inflammation and on the type of CAC model that is used. Therefore, the influences of other upstream signaling that share the MyD88 pathway with TLR4 need to be explored [39-42].

Current treatment for UC, a combination strategy of aminosalicylate compounds, steroids, and biologic agents, has been successful in inducing remission. However, decreasing the risk of colorectal cancer in UC patients has not been satisfied by these strategies. We believe that by blocking some steps of the TLR4-mediated COX-2-PGE_2_ and the AR-EGFR axes is advantageous; while potential adverse effects may have to be taken into account because these pathways are indispensable for the host repair process following mucosal injury. TLR4 antagonists may reduce the incidence of CAC in patients with UC but may delay mucosal healing if colitis is in an active phase [43,44]. A deep understanding of the precise mechanisms regulating mucosal integrity during chronic colitis will provide important insight into the pathogenesis of inflammatory cancers and future development of treatment and prevention strategies.

## References

[1] Fukata M., Chen A., Vamadevan A.S., Cohen J., Breglio K., Krishnareddy S., Hsu D., Xu R., Harpaz N., Dannenberg A.J. (2007). Toll-like receptor-4 promotes the development of colitis-associated colorectal tumors. Gastroenterology.

[2] Hernandez Y., Sotolongo J., Breglio K., Conduah D., Chen A., Xu R., Hsu D., Ungaro R., Hayes L.A., Pastorini C. (2010). The role of prostaglandin E2 (PGE 2) in toll-like receptor 4 (TLR4)-mediated colitis-associated neoplasia. BMC Gastroenterol..

[3] Suzuki R., Kohno H., Sugie S., Tanaka T. (2005). Dose-dependent promoting effect of dextran sodium sulfate on mouse colon carcinogenesis initiated with azoxymethane. Histol. Histopathol..

[4] Suzuki R., Kohno H., Sugie S., Tanaka T. (2004). Sequential observations on the occurrence of preneoplastic and neoplastic lesions in mouse colon treated with azoxymethane and dextran sodium sulfate. Cancer Sci..

[5] McShane L.M., Kulldorff M., Wargovich M.J., Woods C., Purewal M., Freedman L.S., Corle D.K., Burt R.W., Mateski D.J., Lawson M. (1998). An evaluation of rectal mucosal proliferation measure variability sources in the polyp prevention trial: Can we detect informative differences among individuals' proliferation measures amid the noise?. Cancer Epidemiol. Biomarkers Prev..

[6] Noffsinger A.E., Miller M.A., Cusi M.V., Fenoglio-Preiser C.M. (1996). The pattern of cell proliferation in neoplastic and nonneoplastic lesions of ulcerative colitis. Cancer.

[7] Sjoqvist U., Ost A., Lofberg R. (1999). Increased expression of proliferative Ki-67 nuclear antigen is correlated with dysplastic colorectal epithelium in ulcerative colitis. Int. J. Colorectal. Dis..

[8] Coussens L.M., Werb Z. (2002). Inflammation and cancer. Nature.

[9] MacDonald T.T. (1992). Epithelial proliferation in response to gastrointestinal inflammation. Ann. N. Y. Acad. Sci..

[10] Bulut K., Pennartz C., Felderbauer P., Meier J.J., Banasch M., Bulut D., Schmitz F., Schmidt W.E., Hoffmann P. (2008). Glucagon like peptide-2 induces intestinal restitution through VEGF release from subepithelial myofibroblasts. Eur. J. Pharmacol..

[11] Shao J., Sheng G.G., Mifflin R.C., Powell D.W., Sheng H. (2006). Roles of myofibroblasts in prostaglandin E2-stimulated intestinal epithelial proliferation and angiogenesis. Cancer Res..

[12] Pull S.L., Doherty J.M., Mills J.C., Gordon J.I., Stappenbeck T.S. (2005). Activated macrophages are an adaptive element of the colonic epithelial progenitor niche necessary for regenerative responses to injury. Proc. Natl. Acad. Sci. USA.

[13] Brown S.L., Riehl T.E., Walker M.R., Geske M.J., Doherty J.M., Stenson W.F., Stappenbeck T.S. (2007). Myd88-dependent positioning of Ptgs2-expressing stromal cells maintains colonic epithelial proliferation during injury. J. Clin. Invest..

[14] Kundu J.K., Surh Y.J. (2008). Inflammation: gearing the journey to cancer. Mutat. Res..

[15] Ono M. (2008). Molecular links between tumor angiogenesis and inflammation: Inflammatory stimuli of macrophages and cancer cells as targets for therapeutic strategy. Cancer Sci..

[16] Karin M. (2009). NF-kappaB as a critical link between inflammation and cancer. Cold Spring Harb. Perspect. Biol..

[17] Maeda S., Omata M. (2008). Inflammation and cancer: role of nuclear factor-kappaB activation. Cancer Sci..

[18] Greten F.R., Eckmann L., Greten T.F., Park J.M., Li Z.W., Egan L.J., Kagnoff M.F., Karin M. (2004). IKKbeta links inflammation and tumorigenesis in a mouse model of colitis-associated cancer. Cell.

[19] Hayakawa Y., Maeda S., Nakagawa H., Hikiba Y., Shibata W., Sakamoto K., Yanai A., Hirata Y., Ogura K., Muto S. (2009). Effectiveness of IkappaB kinase inhibitors in murine colitis-associated tumorigenesis. J. Gastroenterol..

[20] Karin M., Lin A. (2002). NF-kappaB at the crossroads of life and death. Nat. Immunol..

[21] Fukata M., Chen A., Klepper A., Krishnareddy S., Vamadevan A.S., Thomas L.S., Xu R., Inoue H., Arditi M., Dannenberg A.J., Abreu M.T. (2006). Cox-2 is regulated by Toll-like receptor-4 (TLR4) signaling: Role in proliferation and apoptosis in the intestine. Gastroenterology.

[22] Hausmann M., Kiessling S., Mestermann S., Webb G., Spottl T., Andus T., Scholmerich J., Herfarth H., Ray K., Falk W., Rogler G. (2002). Toll-like receptors 2 and 4 are up-regulated during intestinal inflammation. Gastroenterology.

[23] Cario E., Podolsky D.K. (2000). Differential alteration in intestinal epithelial cell expression of toll-like receptor 3 (TLR3) and TLR4 in inflammatory bowel disease. Infect. Immun..

[24] Szebeni B., Veres G., Dezsofi A., Rusai K., Vannay A., Mraz M., Majorova E., Arato A. (2008). Increased expression of Toll-like receptor (TLR) 2 and TLR4 in the colonic mucosa of children with inflammatory bowel disease. Clin. Exp. Immunol..

[25] Ortega-Cava C.F., Ishihara S., Rumi M.A., Kawashima K., Ishimura N., Kazumori H., Udagawa J., Kadowaki Y., Kinoshita Y. (2003). Strategic compartmentalization of toll-like receptor 4 in the mouse gut. J. Immunol..

[26] Singer, Kawka D.W., Schloemann S., Tessner T., Riehl T., Stenson W.F. (1998). Cyclooxygenase 2 is induced in colonic epithelial cells in inflammatory bowel disease. Gastroenterology.

[27] Agoff S.N., Brentnall T.A., Crispin D.A., Taylor S.L., Raaka S., Haggitt R.C., Reed M.W., Afonina I.A., Rabinovitch P.S., Stevens A.C. (2000). The role of cyclooxygenase 2 in ulcerative colitis-associated neoplasia. Am. J. Pathol..

[28] Wiercinska-Drapalo A., Flisiak R., Prokopowicz D. (1999). Mucosal and plasma prostaglandin E2 in ulcerative colitis. Hepatogastroenterology.

[29] Hsu D., Fukata M., Hernandez Y.G., Sotolongo J.P., Goo T., Maki J., Hayes L.A., Ungaro R.C., Chen A., Breglio K.J. (2009). Toll-like receptor 4 differentially regulates epidermal growth factor-related growth factors in response to intestinal mucosal injury. Lab. Invest..

[30] Brandl K., Sun L., Neppl C., Siggs O.M., Le Gall S.M., Tomisato W., Li X., Du X., Maennel D.N., Blobel C.P., Beutler B. (2010). MyD88 signaling in nonhematopoietic cells protects mice against induced colitis by regulating specific EGF receptor ligands. Proc. Natl. Acad. Sci. USA.

[31] Lee D., Pearsall R.S., Das S., Dey S.K., Godfrey V.L., Threadgill D.W. (2004). Epiregulin is not essential for development of intestinal tumors but is required for protection from intestinal damage. Mol. Cell Biol..

[32] Fukata M., Michelsen K.S., Eri R., Thomas L.S., Hu B., Lukasek K., Nast C.C., Lechago J., Xu R., Naiki Y., Soliman A., Arditi M., Abreu M.T. (2005). Toll-like receptor-4 is required for intestinal response to epithelial injury and limiting bacterial translocation in a murine model of acute colitis. Am. J. Physiol. Gastrointest Liver Physiol..

[33] Nishimura T., Andoh A., Inatomi O., Shioya M., Yagi Y., Tsujikawa T., Fujiyama Y. (2008). Amphiregulin and epiregulin expression in neoplastic and inflammatory lesions in the colon. Oncol. Rep..

[34] Morita H., Nakanishi K., Dohi T., Yasugi E., Oshima M. (1999). Phospholipid turnover in the inflamed intestinal mucosa: Arachidonic acid-rich phosphatidyl/plasmenyl-ethanolamine in the mucosa in inflammatory bowel disease. J. Gastroenterol..

[35] Wallace J.L. (2001). Prostaglandin biology in inflammatory bowel disease. Gastroenterol. Clin. North. Am..

[36] Mutoh M., Takahashi M., Wakabayashi K. (2006). Roles of prostanoids in colon carcinogenesis and their potential targeting for cancer chemoprevention. Curr. Pharm. Design..

[37] Kim E.H., Surh Y.J. (2006). 15-deoxy-Delta12,14-prostaglandin J2 as a potential endogenous regulator of redox-sensitive transcription factors. Biochem. Pharmacol..

[38] Scher J.U., Pillinger M.H. (2005). 15d-PGJ2: The anti-inflammatory prostaglandin?. Clin. Immunol..

[39] Rakoff-Nahoum S., Medzhitov R. (2007). Regulation of spontaneous intestinal tumorigenesis through the adaptor protein MyD88. Science.

[40] Lee S.H., Hu L.L., Gonzalez-Navajas J., Seo G.S., Shen C., Brick J., Herdman S., Varki N., Corr M., Lee J., Raz E. (2010). ERK activation drives intestinal tumorigenesis in Apc(min/+) mice. Nat. Med..

[41] Uronis J.M., Muhlbauer M., Herfarth H.H., Rubinas T.C., Jones G.S., Jobin C. (2009). Modulation of the intestinal microbiota alters colitis-associated colorectal cancer susceptibility. PLoS One.

[42] Salcedo R., Worschech A., Cardone M., Jones Y., Gyulai Z., Dai R.M., Wang E., Ma W., Haines D., O'HUigin C. (2010). MyD88-mediated signaling prevents development of adenocarcinomas of the colon: role of interleukin 18. J. Exp. Med..

[43] Fukata M., Shang L., Santaolalla R., Sotolongo J., Pastorini C., Espana C., Ungaro R., Harpaz N., Cooper H.S., Elson G. (2011). Constitutive activation of epithelial TLR4 augments inflammatory responses to mucosal injury and drives colitis-associated tumorigenesis. Inflamm. Bowel. Dis..

[44] Ungaro R., Fukata M., Hsu D., Hernandez Y., Breglio K., Chen A., Xu R., Sotolongo J., Espana C., Zaias J. (2009). A novel Toll-like receptor 4 antagonist antibody ameliorates inflammation but impairs mucosal healing in murine colitis. Am. J. Physiol. Gastrointest Liver Physiol..

